# Molecular identification of *Mycoplasma synoviae* from breeder chicken flock showing arthritis in Egypt

**DOI:** 10.14202/vetworld.2019.535-541

**Published:** 2019-04-14

**Authors:** Mohamed M. Amer, Hoda M. Mekky, Hanaa S. Fedawy

**Affiliations:** 1 Department of Poultry Diseases, Faculty of Veterinary Medicine, Cairo University, P.O. 12211, Giza, Egypt; 2 Poultry Diseases Department, Veterinary Research Division, National Research Centre, P.O. 12622, Giza, Egypt

**Keywords:** arthritis, *Mycoplasma synoviae* variable lipoprotein hemagglutinin A gene, *Mycoplasma synoviae*, polymerase chain reaction

## Abstract

**Aim:**

Arthritis is one of the most economic problems facing poultry industry worldwide. The study was done to detect possible causes of arthritis in breeder chicken flock with emphasis on molecular identification of *Mycoplasma synoviae* (MS).

**Materials and Methods:**

This study was carried on chicken from broiler breeder flock of 57 weeks’ age in Dakahlia, Egypt, suffered from arthritis with frequently 5-7% decrease in egg production, reduced fertility, and hatchability. Forty blood samples were randomly collected from individual birds in sterile tubes and used for serum separation. Serum samples were tested using serum plate agglutination (SPA) test against colored antigens for *Mycoplasma gallisepticum* (MG), MS, and *Salmonella gallinarum-pullorum* (SGP). On the other hand, 24 joint samples were collected. Of those 24 samples, 12 joint samples were subjected to bacteriological examination, while the other 12 were utilized for molecular diagnosis by polymerase chain reaction (PCR) for MS and avian reovirus (ARV).

**Results:**

SPA test results revealed the presence of antibodies against MG, MS, and SGP in tested sera in rates of 14/40 (35%), 35/40 (87.5%), and 9/40 (22.5%), respectively. Furthermore, 19 bacterial isolates were recognized from joint samples and identified as five *Staphylococcus* spp., nine *Escherichia coli*, three SGP, one *Citrobacter*, and one *Proteus*. The identified Staphylococcal isolates were three coagulase-positive staphylococci (two *Staphylococcus aureus* and one *Staphylococcus hyicus*) and two coagulase-negative staphylococci (one *Staphylococcus epidermidis* and one *Staphylococcus lentus*), while *E. coli* isolate serotypes were 1 O11, 2 O55, 3 O78, 1 O124, 1 O125, and 1 untyped. PCR proved that 12/12 (100%) samples were positive for MS variable lipoprotein hemagglutinin A (*vlhA)* gene, while ARV was not diagnosed in any of the examined samples. Four amplified *vlhA* gene of MS isolates (named MS-2018D1, MS-2018D2, MS-2018D3, and MS-2018D4) was successfully sequenced.

Analysis of phylogenetic tree revealed the presence of 100% identity between each two sequenced isolates(isolates MS-2018D1 and MS-2018D4 and also isolates 2018D2 and MS-2018D3). However, the nucleotide similarity between four isolates was 88.6%. On the other hand, our field isolates MS-2018D1, MS-2018D4, MS-2018D2, and MS-2018D3 showed nucleotide identity with vaccine strain MS-H 98.4%, 98.4%, 88.1%, and 88.1%, respectively. Furthermore, the nucleotide similarities with field strains from Argentina ranged between 87.8% and 98.6%.

**Conclusion:**

Four field isolates of MS were identified in examined broiler breeder flock. A phylogenetic study of these isolates revealed the variation between isolated MS strains and vaccine strain. Therefore, further studies are required for evaluating the vaccine efficacy against the present field isolates of MS. In addition, application of MS immunization of breeder flocks is necessary for proper control of the disease.

## Introduction

Arthritis and tenosynovitis of the hock and stifle joints are the most common skeletal affections in breeder chicken, in addition to spondylitis and osteomyelitis lesions [1]. Arthritis is a worldwide welfare issue in poultry production, caused by many bacterial and viral pathogens. Of those bacterial pathogens, *Mycoplasma synoviae* (MS), *Salmonella* spp., *Escherichia coli*, *Staphylococci*, and *Enterococcus* have been isolated from clinical cases of arthritis [2-5].

As mentioned above, MS is one of the most important bacterial pathogens causing arthritis. It is a worldwide economically important pathogen of poultry, causing respiratory affections, synovitis, abnormalities in eggshell, and drop in production in chickens and turkeys [6-9]. Mostly, MS infections are subclinical with lesions confined to air sac. While in the case of combination with secondary infections, MS becomes systemic and affects the synovial membrane of joints and tendon causing acute and chronic infectious synovitis [10-12]. Birds suffering from infectious synovitis are depressed, reluctant to walk with lameness, and affected hock joints are swollen and hot. The synovial membranes of tendon sheaths become thickened, edematous, with fibrinous exudates accumulation within and around the tendon sheaths [4]. MS is transmitted laterally and vertically, with lateral transmission through direct contact with infected carrier birds and fomites [13]. MS infection in poultry flocks can be diagnosed either serologically, mainly done by serum plate agglutination (SPA) and hemagglutination inhibition, or through direct microbial isolation and identification that represent the gold standard, but time-consuming due to long incubation and fastidious nature of mycoplasmas [6]. Recently, molecular diagnosis with a polymerase chain reaction (PCR) is commonly used [14-16]. SPA test is quick, inexpensive, and sensitive test used for serological screening for *Mycoplasma* spp. and *Salmonella* spp. [17]. The *vlhA* gene of MS encodes for hemagglutinin and other immunodominant membrane proteins involved in colonization, antigenic variations, and virulence. Sequence analysis of the MS *vlhA* gene region has been used for the classification of strains and epidemiological studies and should be regarded as a preliminary typing method [18-20].

Concerning viral pathogens involved in arthritis, avian reovirus (ARV) has been recognized to be responsible for many cases of chicken viral arthritis/tenosynovitis among layer and broiler chickens in recent years [21]. ARV infection is usually present in poultry flocks without clinical manifestations. However, it may be involved in several disease syndromes including viral arthritis or tenosynovitis, respiratory disease, enteric disease, stunting syndrome, immunosuppression, and malabsorption syndrome. Viral arthritis/tenosynovitis is the most important in broiler breeder chickens. ARV can induce arthritis or tenosynovitis either alone or in combination with other pathogens as MS, *Staphylococcus* spp., and *E. coli* [22-24]. The ARV genome consists of 10 segments of double-stranded RNA: Four small (S1, S2, S3, and S4), three medium (M1, M2, and M3), and three large (L1, L2, and L3) [22]. Diagnosis of ARV is based on the detection of the virus in clinically affected joints which can be achieved by molecular techniques [25-30].

This study aimed to detect the possible pathogens involved in field cases of arthritis from breeder chicken flock with special reference to molecular identification of MS and ARV.

## Materials and Methods

### Ethical approval

This study was approved by Ethical Committee for Medical Research at the National Research Centre, Egypt and in accordance with local laws and regulations.

### Field samples

This study was carried out on broiler breeder flock of 57 weeks old located in Dakahlia, Egypt. Birds suffered from arthritis with a history of 5-7% decrease in egg production and decreased fertility and hatchability. Blood and joint samples were collected as follows:

- Forty blood samples were collected randomly from individual birds with arthritis in sterile tubes and used for serum separation by centrifugation at 3000 rpm for 20 min. Sera were tested using SPA test for *Mycoplasma gallisepticum* (MG) and MS as well as *Salmonella gallinarum-pullorum* (SGP) against colored antigens obtained from Intervet Co., Boxmeer, Netherlands. Serum agglutination tests were carried out according to the National Poultry Improvement Plan [31].
- Twenty-four joint samples (synovial fluid) were collected under aseptic conditions after disinfection of joint externally with alcohol. Due to the shortage and insufficiency of financial resources, samples were divided into two groups (each of 12 samples). Samples of the first group were subjected to bacteriological examination, while in second group, each sample was subsequently subdivided into two parts. One part was forwarded for reverse transcription PCR (RT-PCR) application for ARV diagnosis, and the other part was directed for PCR application for MS diagnosis.

### Bacteriological examination

Aseptically collected joint samples in the first group, subjected to bacteriological examination, were inoculated into tryptic soy broth and incubated at 37°C for 18 h, then subcultured on to 5% sheep blood agar base, *Salmonella Shigell*a Agar, MacConkey agar, Eosin methylene blue agar, Mannitol salt agar, nutrient agar, and tryptic soy agar plates, and incubated at 37°C for 24-48 h. The obtained bacterial growth was purified and examined for colonial morphology and staining characteristics [32] and subjected to biochemical identification [33-35]. *E. coli* and *Salmonella* spp. strains were serotyped using slide agglutination test against polyvalent and monovalent standard antisera obtained from Behring Werke Institute, Germany, using methods of Neville and Brgant [36] and Lee and Arp [37].

### PCR for MS and ARV

#### MS

Sample preparation

Aseptically collected joint samples were inoculated in pleuropneumonia-like organism (PPLO) broth. The inoculated media were incubated at 37°C in humid CO_2_ incubator for 24 h [38]. The propagated bacteria were harvested by centrifugation at 14,000 g for 10 min, and microbial pellet was resuspended in 300 μl PBS for DNA extraction.

DNA extraction

The suspended bacterial pellet was subjected to DNA extraction using the QIAamp DNA Mini kit (Qiagen, Germany, GmbH) with modifications from the manufacturer’s recommendations. Briefly, 200 µl of the sample suspension was incubated with 10 µl of proteinase K and 200 µl of lysis buffer at 56°C for 10 min. Then, 200 µl of 100% ethanol was added to the lysate. The precipitated nucleic acid was then washed and centrifuged. Extracted DNA was eluted with 100 µl of elution buffer provided in the kit.

PCR amplification

The PCR reaction mix was performed in a total volume of 25 μl per sample, containing 12.5 µl of EmeraldAmp Max PCR Master Mix (Takara, Japan), 1 µl of each primer of 20 pmol concentrations, 4.5 µl of deionized distilled water, and 6 µl of DNA template. The reaction was performed in an Applied Biosystems 2720 Thermal Cycler as follows: 5 min at 94°C, followed by 35 cycles of 30 s at 94°C, 40 s at 55°C, and 45 s at 72°C, with a final extension cycle of 10 min at 72°C, producing a fragment of 396 bp. Oligonucleotide primers supplied from Metabion, Germany, are listed in Table-1. The positive control was a commercially available MS isolate provided by Charles River Laboratories, Inc., USA, Batch 25121227A.

**Table-1:** Oligonucleotides primers sequences, target genes, and amplicon sizes used in PCR.

Target agent	Target gene	Primer sequence (5’- 3’)	Amplified segment (bp)	Reference
MS	*vlhA*	F: GATGCGTAAAATAAAAGGAT	396	Hong *et al*. [18]
		R: GCTTCTGTTGTAGTTGCTTC		
ARV	S2	REO-F: CCC ATG GCA ACG ATT TC	399	Bruhn *et al*. [26]
		REO-R: TTC GGC CAG GTC TCA AC		

PCR=Polymerase chain reaction, ARV=Avian reovirus, *vlhA=*Variable lipoprotein hemagglutinin A, MS=*Mycoplasma synoviae*

#### ARV

Sample preparation

Twelve joint samples were subjected to centrifugation at 3000× g for 5 min and then filtrated through a sterile 0.22-μm membrane filter. The filtered supernatants were collected in a 1.5-ml sterile RNase- and DNase-free microtube and stored at −20°C until use for RNA extraction.

RNA extraction

Filtered joint samples were subjected to RNA extraction using the QIAamp Viral RNA Mini Kit (Qiagen, Germany, GmbH). Briefly, 140 µl of the filtered sample was incubated with 560 µl of AVL lysis buffer containing 5.6 µl of carrier RNA at room temperature for 10 min. After incubation, 560 µl of 100% ethanol was added to the lysate. The samples were then washed and centrifuged following the manufacturer’s recommendations. Nucleic acid was eluted with 60 µl of elution buffer provided in the kit.

RT-PCR amplification

Amplification was performed in a total volume of 25 μl containing 12.5 µl of QuantiTect Probe RT-PCR Buffer (Qiagen, GmbH), 1 µl of each primer of 20 pmol concentration, 0.25 µl of rt-enzyme, 4.25 µl of water, and 6 µl of RNA template. The reaction was performed in a Biometra Thermal Cycler. Thermal conditions consisted of RT at 50°C for 30 min and then primary denaturation at 95°C for 5 min. These steps were followed by 35 cycles of 94°C for 30 s, 55°C for 45 s, and 72°C for 45 s. The last step was done at 72°C for 10 min, producing a fragment of 399 bp. Oligonucleotide primers supplied from Metabion, Germany, are listed in Table-1. The positive control was ARV field sample previously confirmed (to be positive for S2 gene) in reference laboratory for veterinary quality control on poultry production, Animal Health Research Institute.

### MS and ARV analysis of the PCR products

The products of PCR were separated by electrophoresis on 1.5% agarose gel (Applichem, Germany, GmbH) in 1× TBE buffer at room temperature using gradients of 5 V/cm. For gel analysis, 15 µl of the products were loaded in each gel slot. A 100 bp DNA ladder (Qiagen, Germany, GmbH) was used to determine the fragment sizes. The gel was photographed by a gel documentation system (Alpha Innotech, Biometra), and the data were analyzed through computer software.

### MS vlhA gene sequencing and phylogeny

Four PCR products of *vlhA* gene of MS isolates, named MS-2018D1, MS-2018D2, MS-2018D3, and MS-2018D4, were purified using QIAquick PCR Product Extraction Kit (Qiagen, Valencia). The sequence reaction was done by Bigdye Terminator V3.1 Cycle Sequencing Kit (Perkin-Elmer), and then, it was purified using Centrisep spin column. DNA sequences were performed by Applied Biosystems 3130 Genetic Analyzer (Hitachi, Japan). To establish sequence identity to GenBank accessions, a BLAST^®^ analysis (Basic Local Alignment Search Tool) [39] was initially performed. The phylogenetic tree was obtained by the MegAlign module of Lasergene DNAStar [40] and phylogenetic analyses were conducted with published data of other 22 isolates (Table-2) based on maximum likelihood, neighbor-joining, and maximum parsimony in MEGA6 [41].

**Table-2:** Published MS sequences of *vlhA* used for multiple alignment analysis.

MS partial amplification of *vlhA* gene	GenBank accession no.	Isolate name	Strain type	Country of origin
1	CP021129.1	MS-H	Vaccine strain	Australia
2	CP012624.1	86079-7NS	The parent strain of temperature-sensitive MS-H vaccine	Australia: Victoria
3	KP704286.1	MS-H	Vaccine strain	Australia
4	CP011096.1	WVU1853	Field strain	USA: West Virginia
5	FJ890931.1	WVU 1853; ATCC 25204	Field strain	Tunisia
6	AF035624.1	WVU-1853	Field strain	Australia
7	HQ326479.1	WVU1853	Field strain	USA: West Virginia
8	GU084388.1	ULB02/T6	Field strain	Slovenia
9	GU084387.1	T65M/T3K2	Field strain	Slovenia
10	GU084386.1	ULB02/OV6K1	Field strain	Slovenia
11	GU084385.1	T65M/T3K1	Field strain	Slovenia
12	GU084384.1	T68W/IP2K	Field strain	Slovenia
13	GU084383.1	T68W/IT2B	Field strain	Slovenia
14	GU084382.1	T68W/IT2A	Field strain	Slovenia
15	GU451303.1	T68W/IT1A	Field strain	Slovenia
16	HQ326477.1	MS173	Field strain	Argentina
17	HQ326476.1	MS117	Field strain	Argentina
18	AF085698.1	WUV-1853	Field strain	Australia
19	AF085697.1	WVU-1853	Field strain	Australia
20	AF464937.1	MS-H	Vaccine strain	Australia
21	AF464938.1	MS-H	Vaccine strain	Australia
22	AF464936.1	MS-H	Vaccine strain	Australia
23	MH605097*	MS-2018D1	Field strain	Egypt
24	MH605098*	MS-2018D4	Field strain	Egypt
25	MH605099*	MS-2018D2	Field strain	Egypt
26	MH605100*	MS-2018D3	Field strain	Egypt

* Indicates MS field isolates of the current study. *vlhA=*Variable lipoprotein hemagglutinin A, MS=*Mycoplasma synoviae*

## Results and Discussion

Locomotor disorders are a terrible challenge facing the poultry industry and represent a major economic and welfare problem. The most important locomotor affection is septic arthritis, which means a microbial infection of one or more joints [42]. In Egypt, arthritis in broiler breeders causes high economic losses as a result of lameness accompanied by an inability to feed, resulting in loss of weight and even mortalities.

Multiple organisms can cause septic arthritis in poultry. The most important organisms are *Staphylococcus* spp., *E. coli*, *Salmonella* spp., *Enterococcus* spp., MS, in addition to ARV [1,4].

MS is an important poultry pathogen, causing infectious synovitis and respiratory disease. MS infection can be diagnosed serologically by SPA test, which is considered as the initial qualitative screening test used for monitoring antibodies against the pathogen. Besides, it is also used for the screening of other pathogens such as MG and SGP in poultry flocks. The test is rapid, inexpensive with efficient sensitivity but low specificity [14,24].

In our study, the tested sera by SPA test showed positive results for MG, MS, and SGP in rates of 14/40 (35%), 35/40 (87.5%), and 9/40 (22.5 %), respectively. The obtained results indicate the circulation of these bacteria among birds of the tested flock. These results fairly close to that obtained by Amer and El-Ghany [43] who reported that the prevalence of MG and MS was 44.35% and 63%, respectively, in breeder chicken flocks by SPA test but differ than that obtained by Silva *et al*. [44] who reported negative results for *Salmonella pulloru*m, *Salmonella gallinarum*, and MG and only positive results (2/135, 1.48%) for MS using SPA test.

Bacteriological examination of joint samples revealed identification of 19 bacterial isolates through morphological and biochemical characterization of grown bacteria. The isolated bacteria were five *Staphylococcus* spp. isolates, nine *E. coli* isolates, three SGP isolates, one *Citrobacter*, and one *Proteus*. The *Staphylococci* isolates were identified into three coagulase-positive *Staphylococcus* (two *Staphylococcus aureus* and one *Staphylococcus hyicus*) and two coagulase-negative staphylococci (one *Staphylococcus epidermidis* and one *Staphylococcus lentus*). *E. coli* isolates were serotyped into one O11, two O55, three O78, one O124, one O125, and one untyped. These results are similar to those reported by Nazia *et al*. [42], Rasheed [45], Braga *et al*. [46], and Tawfik *et al*. [47] who isolated *E. coli, S. aureus, Salmonella Enterica, Pseudomonas aeruginosa*, and *Erysipelothrix rhusiopathiae* from hock joint.

The study targeted MS and ARV as major infectious causes of leg problems emerging at the broiler breeder level and also characterized by their vertical transmission [24]. The nature of MS is being a fastidious organism needs special nutritional requirements, thus its culturing and isolation is not preferred for flock screening due to its difficulty and time-consuming. To control the infection and subsequently limit economic losses in poultry industry, obstacles of traditional isolation of MS were overcome through the application of early, rapid, and accurate diagnostic methods as PCR for the detection and diagnosis [19,48,49].

In our study, PCR for MS identification was applied after 24 h of cultivation in PPLO broth as application of PCR on MS inoculated and incubated PPLO broth 24-48 h was of a higher rate than those applied directly on joint samples. This might be due to MS propagation which improves the detection limit of PCR. Moreover, more reduction of PCR inhibitor was from PPLO broth than from tissue samples [38,50,51].

**Figure-1:**
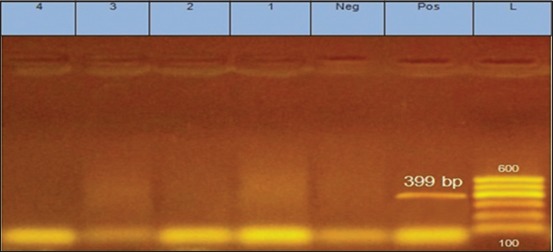
Amplified S2 gene of avian reovirus. Lane L = 100 bp marker; Lanes 1-4 = Examined samples; Lane Neg = Negative control; Lane Pos = Positive control.

**Figure-2:**
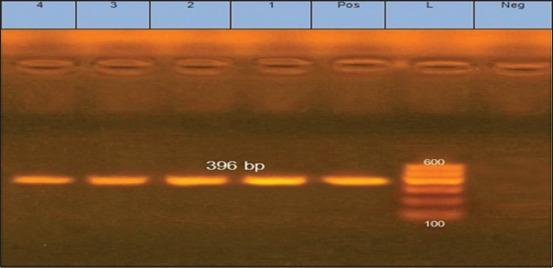
Amplified *vlhA* gene of *Mycoplasma synoviae*. Lane L = 100 bp marker; Lanes 1-4 = Examined samples; Lane Neg = Negative control; Lane Pos = Positive control.

The RT-PCR for S2 gene of ARV was negative for all examined samples as shown in Figure-1. On the other hand, PCR for *vlhA* gene of MS revealed the presence of 12/12 (100%) positive samples as shown in Figure-2. Our results were opposite to those obtained by Souza *et al*. [30] who found that all examined samples were positive for ARV and negative for MS and MG by PCR. Furthermore, the results were different from that obtained by Reck *et al*. [52] who detected MS in 61.79% (131/212), ARV in 25.47% (54/212), and both MS and ARV in 18.86% (40/212) of examined samples of tibiotarsal articulation of broiler chickens by multiplex PCR.

The successfully amplified *vlhA* gene of four MS isolates, named MS-2018D1, MS-2018D2, MS-2018D3, and MS-2018D4, was sequenced and submitted to NCBI GenBank and the obtained accession numbers are MH605097, MH605099, MH605100, and MH605098, respectively.

As this broiler breeder flock is not vaccinated against MS, so alignment of amplified products of four field isolates with vaccine strain (MS-H) and other published *vlhA* sequence (Table-2) was applied. Phylogenetic tree analysis according to sequences of *vlhA* gene showed 100% homology between sequenced isolates MS-2018D1 and MS-2018D4 and also between isolates MS-2018D2 and MS-2018D3 of the current study; this may be due to the same source of infection. While isolates MS-2018D1 and MS-2018D4 showed the same nucleotide similarity (88.6%) when compared with isolates MS-2018D2 and MS-2018D3.

**Figure-3:**
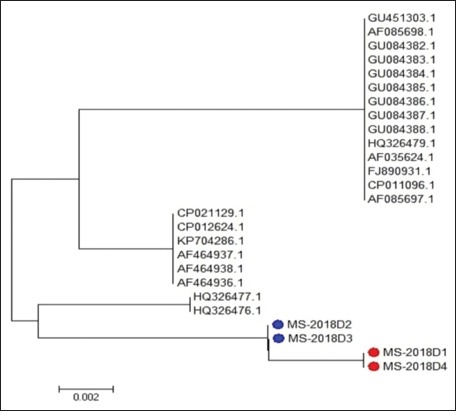
Phylogenetic tree of *Mycoplasma synoviae* based on the nucleotide sequence of *vlhA* gene. Branched distances correspond to sequence divergence.

**Figure-4:**
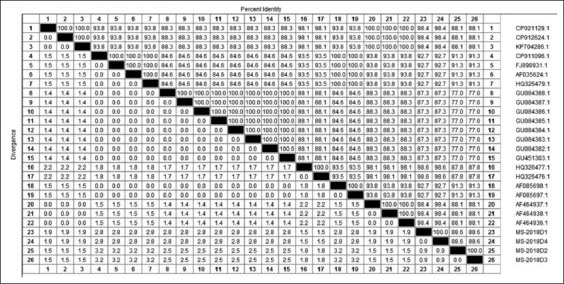
Percentage of nucleotide identities for the *vlhA* genes of four MS strains named MS-2018D1, MS-2018D2, MS-2018D3, and MS-2018D4 as compared with 22 sequences published in GenBank.

Egyptian field isolates MS-2018D1, MS-2018D4, MS-2018D2, and MS-2018D3 had 98.4%, 98.4%, 88.1%, and 88.1% nucleotide identity with vaccine strain MS-H, respectively. Furthermore, the four Egyptian isolates had similarities 87.8-98.6% with field strains from Argentina, while the identity was 77.0-92.7% with other field isolates from Tunisia, Australia, USA, and Slovenia (Figures-3 and 4).

## Conclusion

Four field isolates of MS were identified in the examined broiler breeder flock. A phylogenetic study of these isolates showed the presence of variation between isolated field MS strains and vaccine strain. Hence, further studies are recommended to detect the vaccine efficacy against the present field isolates of MS. Implementation of proper MS immunization program is important for breeder flocks in order to control the disease.

## Authors’ Contributions

MMA designed, planned this study, drafted, and revised the manuscript. HMM and HSF shared in samples collection and performed the tests, manuscript writing, and data analysis. All authors read and approved the final manuscript.
